# Peritonitis Caused by Rupture of Infected Retroperitoneal Teratoma

**Published:** 2012-03-01

**Authors:** Yogesh Kumar Sarin

**Affiliations:** Department of Pediatric Surgery Maulana Azad Medical College New Delhi-110002, INDIA

**Keywords:** Retroperitoneal teratoma, Peritonitis, Infected teratoma, Mature teratoma

## Abstract

Retroperitoneal teratomas are usually asymptomatic, though there have been isolated reports of retroperitoneal teratomas presenting as intra-abdominal abscesses and peritonitis in adults. A 7-year-old girl who had presented with acute abdomen due to ruptured retroperitoneal teratoma is reported.

## INTRODUCTION

Retroperitoneal teratomas (RPTs) are uncommon tumors representing about 5% of all teratomas [1]. Majority of them are benign. They are typically asymptomatic; but when symptoms do occur due to enormity of their size, patients will present with only abdominal distension or a palpable mass on physical examination [2]. RPTs resulting in chemical peritonitis or localized abscess and presenting as acute abdomen have rarely been described in adults [3-6]. RPT presenting as acute abdomen has been even rarer in pediatric patients. An extensive literature search could reveal only 2 cases hitherto [7,8]. We report here another case of ruptured retroperitoneal teratoma that resulted in chemical peritonitis. 

## CASE REPORT

A 7-year-old girl presented with abdominal mass that was noted at birth. She had abdominal pain and recurrent febrile episodes for the last 6 months that had worsened a week before presentation. On examination, she was febrile with generalized abdominal tenderness. A large well-defined, firm, fixed, tender mass, having bosselated surface and measuring 15 cms in diameter occupied entire left half of her abdomen. The fingers could be insinuated between the mass and left costal margin above and the mass and the pelvic brim below. Leukocyte count was 14,000/ mm3. Biochemical parameters were normal. Abdominal roentgenogram showed a soft tissue shadow occupying the left half of the abdomen displacing the stomach up and the bowel loops to the right. There were extensive areas of calcification (Fig. 1). Chest x ray was normal. Abdominal ultrasound revealed a large heterogeneous retroperitoneal mass pushing the left kidney and the ureter with mild to moderate left hydronephrosis. CT scan abdomen showed a well-defined retroperitoneal mass measuring 14cm x 10cm x 9cm in the left half of the abdomen having mixed density, septations, calcifications and teeth-like structures (Fig. 2,3). The mass displaced the left kidney posteriorly and cranially, the sigmoid colon anteriorly, and aorta and inferior vena cava to the right. The serum alpha fetoprotein levels were within normal range. The diagnosis of infected retroperitoneal benign teratoma was made.

**Figure:**
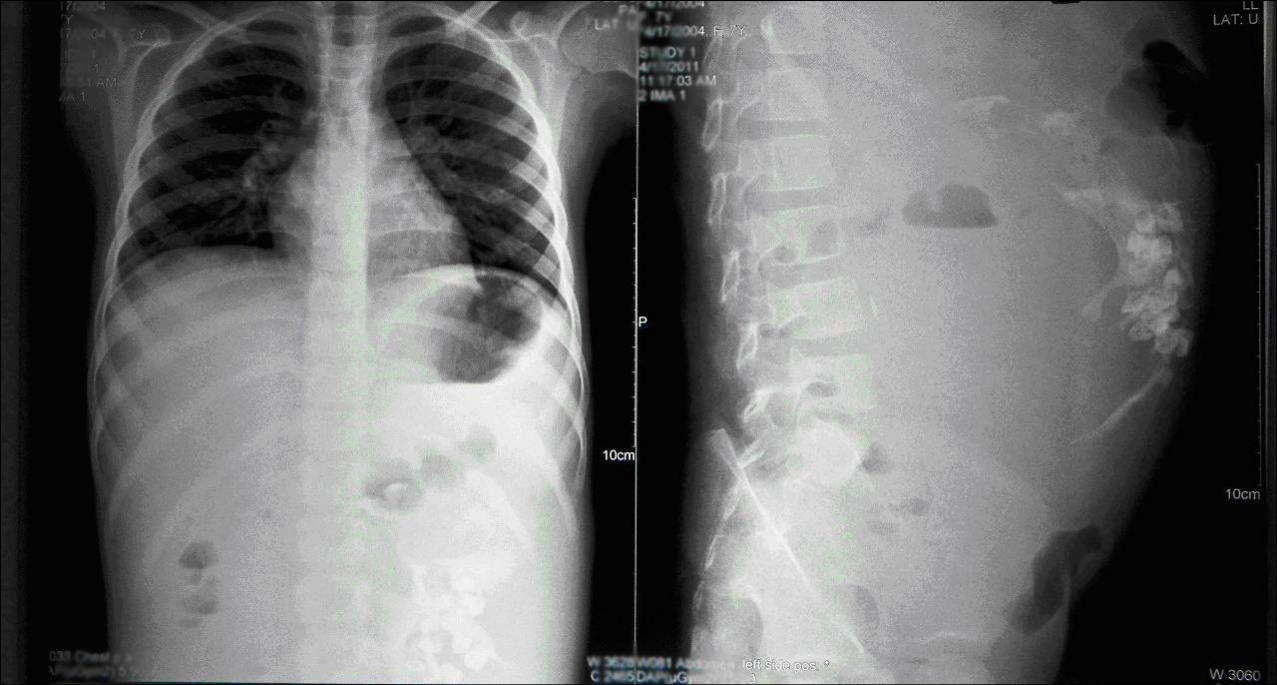
Figure 1: Abdominal roentgenograms showing soft-tissue mass displacing gut and having calcifications.

**Figure:**
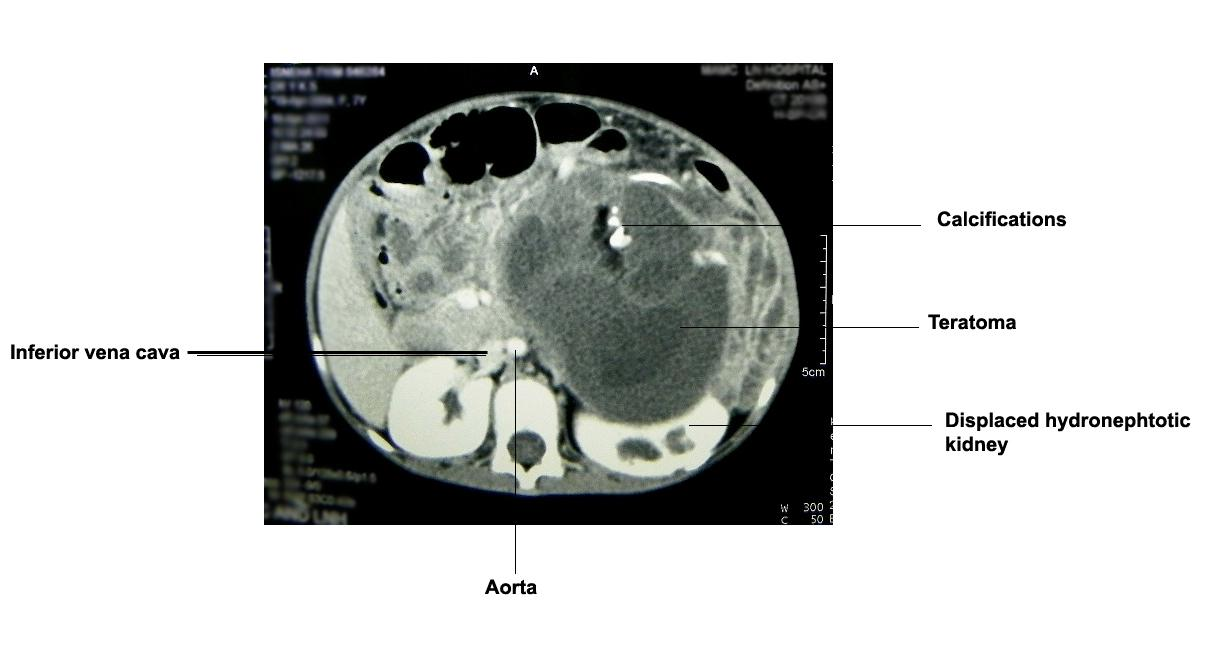
Figure 2: Abdominal CT scan showing well circumscribed mass occupying left half of the abdomen, having septations and calcifications and displacement of gut and major vessels.

**Figure:**
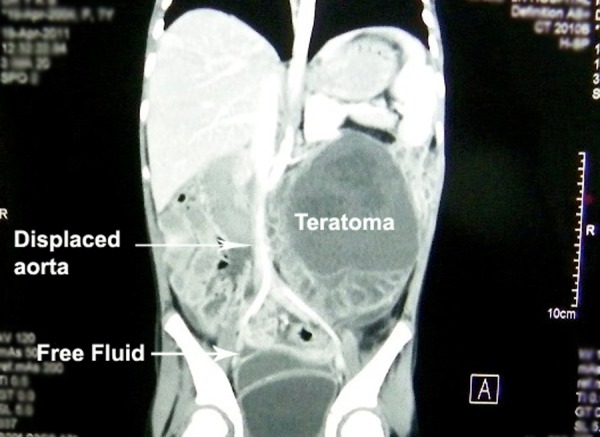
Figure 3: Abdominal CT scan showing well circumcised mass occupying left half of the abdomen, having septations and calcifications and displacing gut and major vessels.

At operation a small amount of thick turbid came out. There were inter-loop pockets. The thick capsule of the tumor was found breached at 2 places. The overlying sigmoid colon was firmly adherent to the tumor capsule. The aorta, inferior vena cava and the mesenteric vessels were pushed to the right and did not pose any risk to the dissection. Both ovaries were normal.

Excision of the large tumor necessitated resection and anastomosis of the sigmoid colon. Though the tumor could be removed in toto, there was gross spillage intra-operatively. Few para-aortic lymph nodes were sampled. The resected specimen had variegated appearance and there was evidence of cartilage, teeth, and hairs (Fig. 4).

**Figure:**
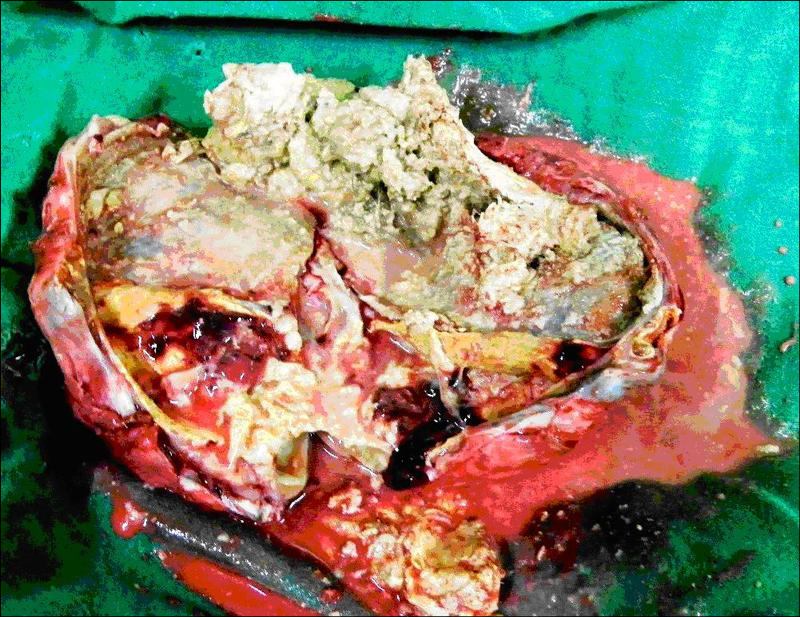
Figure 4: Excised specimen showing calcification, hair and other features suggestive of mature teratoma.

The patient did well post-operatively and was discharged on 15th postoperative day. The biopsy was reported as mature cystic teratoma (dermoid cyst) with evidence of extensive xanthogranulomatous reaction to keratin. The para-aortic lymph nodes had only reactive changes. She has been on close follow up since last 6 months and has been doing well. 

## DISCUSSION

Retroperitoneal teratomas are rare, representing only 1% to 11% of primary retroperitoneal neoplasms [8]. They typically present as asymptomatic abdominal mass but can grow to enormous size. There are only 2 children reported hitherto who had presented with acute abdomen. Pontinen et al almost half century ago, had reported a retroperitoneal teratoma in a three year old girl that simulated an acute appendicitis [7]. Nguyen et al had recently described a 9-year-old girl who presented with an acute abdomen because of an abdominal abscess that was treated with surgical drainage and antibiotics [8]. Fifteen years later, she had a recurrence of symptoms and the abscess was ultimately recognized to be an infected retroperitoneal teratoma. Though the diagnosis of infected retroperitoneal teratoma was made at the age of 24 years, this teratoma must have been present since birth and got missed at the earlier presentation in the childhood.

Most of the retroperitoneal tumors in childhood are cystic and benign [2]. Spontaneous rupture of cystic retroperitoneal teratomas is a rare occurrence probably because of the thick encasing capsule. Taking analogy from cystic ovarian teratomas, two clinical presentations could be associated with such intraperitoneal rupture of benign cystic teratomas [9]. The first is acute peritonitis caused by the sudden rupture of tumor contents, which may occur spontaneously or more commonly in association with torsion, trauma, infection, or labour. The second presentation is chronic granulomatous peritonitis resulting from a chronically leaking dermoid, which can be characterized by multiple small white peritoneal implants, dense adhesions, and variable ascitis that simulate carcinomatosis or tuberculous peritonitis. The latter is the more common presentation in case of cystic ovarian teratomas [9].

v CT scan is considered as better radiological investigation than ultrasonography for the diagnosis of RPTs [10]. MRI has been also used recently. At CT, a mature RPT manifests as a complex mass containing a well-circumscribed fluid component, adipose tissue, and calcification [10]. The presence of hypoattenuating fat within the cyst and the presence of calcifications in the cyst wall are considered highly suggestive of cystic RPT [10]. At CT, the presence of fat-fluid levels in the peritoneum has been quoted as a reliable sign of intraperitoneal rupture of abdominal teratoma and subsequent chemical peritonitis [3]. However, the diagnosis of rupture of RPT is usually made at operation.

The operative management of RPTs, especially those with rupture, may be complex and challenging. Despite their benign nature, the lesions can attenuate and surround major vessels, making resection difficult. Preoperative imaging has been known to be offer limited help in demonstrating the position of the major vessels [11]. In particular, the veins may be effaced. Excision of ruptured RPT in our case was also a formidable surgical exercise.

In conclusion, rupture of RPT is an extremely rare phenomenon. It may be difficult to make a preoperative diagnosis and the surgical excision could be a challenging task.

## Footnotes

**Source of Support:** Nil

**Conflict of Interest:** None declared

## References

[1] ( 1976). Grosfeld JL, Ballantine TV, Lowe D, Baehner RL. Benign and malignant teratomas in children: Analysis of 85 patients. Surgery.

[2] ( 2006). Chaudhary A, Misra S, Wakhlu A, Tandon RK, Wakhlu AK. Retroperitoneal teratomas in children. Indian J Pediatr.

[3] ( 1990). Ferrero A, Cacspedes M, Cantarero JM, Arenas A, Pamplona M. Peritonitis due to rupture of retroperitoneal teratoma: computed tomography diagnosis. Gastrointest Radiol.

[4] ( 2005). Talwar N, Andley M, Ravi B, Kumar A. Subhepatic abscess in pregnancy- an unusual presentation of infected primary retroperitoneal teratoma. Acta Obstet Gynecol Scand.

[5] ( 2000). Pandya JS, Pai MV, Muchhala S. Retroperitoneal teratoma presenting as acute abdomen in an elderly person. Indian J Gastroenterol.

[6] ( 2010). Li F, Munireddy S, Jiang L, Cheng N, Mao H, Pawlik TM. Infected primary retroperitoneal teratoma presenting as a subhepatic abscess in a postpartum woman. Am J Surg.

[7] ( 1962). Pontinen PJ, Taulaniemi E. Retroperitoneal teratoma simulating an acute appendicitis in a three year old girl. Ann Chir Gynaecol Fenn.

[8] ( 2007). Nguyen CT, Kratovil T, Edwards MJ. Retroperitoneal teratoma presenting as an abscess in childhood. J Pediatr Surg.

[9] ( 1975). Pantoja E, Noy MA, Axtmayer RW, Colon FE, Pelegrina I. Ovarian dermoids and their complications: comprehensive historical review. Obstet Gynecol Surg.

[10] ( 1991). Jeffrey RB Jr. Imaging of the peritoneal cavity. Curr Opin Radiol.

[11] ( 2008). ones NM, Kiely EM. Retroperitoneal teratomas-potential for surgical misadventure. J Pediatr Surg.

